# Ants, *Cataglyphis cursor*, Use Precisely Directed Rescue Behavior to Free Entrapped Relatives

**DOI:** 10.1371/journal.pone.0006573

**Published:** 2009-08-12

**Authors:** Elise Nowbahari, Alexandra Scohier, Jean-Luc Durand, Karen L. Hollis

**Affiliations:** 1 Laboratoire d'Éthologie Expérimentale et Comparée EA 4443, Université Paris Nord, Villetaneuse, France; 2 Interdisciplinary Program in Neuroscience & Behavior, Mount Holyoke College, South Hadley, Massachusetts, United States of America; University of Utah, United States of America

## Abstract

Although helping behavior is ubiquitous throughout the animal kingdom, actual rescue activity is particularly rare. Nonetheless, here we report the first experimental evidence that ants, *Cataglyphis cursor*, use precisely directed rescue behavior to free entrapped victims; equally important, they carefully discriminate between individuals in distress, offering aid only to nestmates. Our experiments simulate a natural situation, which we often observed in the field when collecting *Catagyphis* ants, causing sand to collapse in the process. Using a novel experimental technique that binds victims experimentally, we observed the behavior of separate, randomly chosen groups of 5 *C. cursor* nestmates under one of six conditions. In five of these conditions, a test stimulus (the “victim”) was ensnared with nylon thread and held partially beneath the sand. The test stimulus was either (1) an individual from the same colony; (2) an individual from a different colony of *C cursor*; (3) an ant from a different ant species; (4) a common prey item; or, (5) a motionless (chilled) nestmate. In the final condition, the test stimulus (6) consisted of the empty snare apparatus. Our results demonstrate that ants are able to recognize what, exactly, holds their relative in place and direct their behavior to that object, the snare, in particular. They begin by excavating sand, which exposes the nylon snare, transporting sand away from it, and then biting at the snare itself. Snare biting, a behavior never before reported in the literature, demonstrates that rescue behavior is far more sophisticated, exact and complexly organized than the simple forms of helping behavior already known, namely limb pulling and sand digging. That is, limb pulling and sand digging could be released directly by a chemical call for help and thus result from a very simple mechanism. However, it's difficult to see how this same releasing mechanism could guide rescuers to the precise location of the nylon thread, and enable them to target their bites to the thread itself.

## Introduction

Despite the fact that numerous forms of helping behavior have been observed in countless vertebrate species [1], actual rescue of one animal by another, even a conspecific, is extremely rare. In the earliest, often-cited example of vertebrate rescue behavior, dolphins assisted injured conspecifics by supporting them to the sea surface so that the victims could breathe more easily [2]. Surprisingly, however, the first published evidence of rescue behavior in coalition-forming capuchin monkeys, animals well-known for their helping behavior, appeared only three years ago and was an observational report of a single interaction [3].

In ants, however, invertebrates well known for their highly integrated and complex cooperative behavior, anecdotes of a simple form of rescue behavior, namely sand digging, was described as early as 1874 [4]. Subsequent reports of digging behavior did not re-appear until the mid-1900s [5]–[11]; however, many of these authors described digging as a simple alarm reaction, not rescue per se. Recently, rescue behavior has been reported in *Formica* workers entrapped in an antlion pit [12], a common predator of many ant species [13]. Not only digging, but also limb pulling behaviors were observed; however, both behavioral patterns appeared to be directed toward any conspecific. In another recent paper [14], ants demonstrated what the authors aptly termed “cooperative self-defense”. That is, when attacked by driver ants, victimized *Pachycondyla analis* ants engage in counterattack behavior; however, this counterattack behavior appears to be directed toward all driver ants, not only to attackers clinging to nestmates' bodies, but also to driver ants that have not yet attacked. In all other studies to date, the rescue behavior was not experimentally studied and the effect of relatives on rescue behavior remains a mystery – surprisingly so given the important explanatory power of kinship in countless other forms of cooperative behavior [15], [16], as well as newer predictive models of cooperation and altruism [17]. Indeed, knowledge of kinship relations has revolutionized the field of behavioral ecology [18]. We therefore studied whether the rescue behavior that we observed in the field when collecting *C. cursor* ants would be delivered indiscriminately to all ants in close proximity, only to members of the same *C. cursor* species, or only to nestmates – a question that, at its core, addresses whether the “call-for-help” is species-specific or is unique to each ant colony. Using an artificial nylon snare that simulated a situation in which ants become entrapped by collapsing sand and debris, we systematically varied the relationship between victims and rescuers to determine the specificity of a potential victim's ability to elicit rescue attempts, as well as different strategies of rescuers to free them.

## Results

The behavior of separate, randomly chosen groups of 5 *C. cursor* nestmates (the potential “rescuers”) was observed under one of five conditions in which a test stimulus (the “victim”) was ensnared and held partially beneath the sand, namely (1) a *C. cursor* nestmate/relative from the same colony as the potential rescuers (homocolonial test), (2) a *C. cursor* individual from a different colony (heterocolonial test), (3) an ant from a sympatric (living in close proximity), unrelated species, *Camponotus aethiops* (heterospecific test), (4) a larval cricket (prey test), and (5) an ensnared motionless (chilled) nestmate. A final test (6) consisted of an empty snare apparatus. The snare consisted of a nylon thread, wrapped around the victim's pedicel in conditions 1–5, and secured to a 1-cm-diameter round of filter paper. In all conditions the filter paper was concealed beneath the sand. In conditions 1–5, the head, antennae and thorax were visible above the sand and the victim could move these body parts freely; in condition 6, only the nylon snare was visible. Each test stimulus was left in place for 7 minutes and videotaped for later analysis. For each of these six test conditions, in which we varied the nature of the test stimulus victim, we conducted 9 independent observations, namely 3 separate samples from each of 3 different *C. cursor* colonies. Thus, we conducted a total of 54 tests. For each test, the group of 5 potential rescuer ants constituted a single statistical unit. That is, potential ant rescuers were marked individually with a distinct color, enabling us to record the duration of each behavior separately for each ant; however, we then added the duration data across the 5 ants and analyzed the total duration.

Across the 54 different tests, 8 distinct behavior patterns emerged, 4 of which were different kinds of rescue behavior while the remaining 4 were characteristic forms of aggressive behavior (see Table S1). Remarkably, only active nestmates (homocolonial test, condition 1) evoked any form of rescue behavior, and they did so in each of the 9 independent homocolonial tests. Rescue behavior never was observed in any of the remaining 45 tests, either with live test individuals – i.e., heterocolonial ants (condition 2), heterospecific ants (condition 3), prey stimuli (condition 4), or with an ensnared motionless (chilled) nestmate (condition 5) – or an empty snare apparatus (condition 6). As Figure S1 illustrates, rescue attempts consisted of digging sand in the area of the ensnared nestmate, transporting particles of sand at least 5 mm (and as far as 2 cm) away from the snare, pulling the limbs of the ensnared nestmate (but never the antennae, highly sensitive appendages that could be injured easily) and, most important, biting precisely at the nylon snare that entrapped a relative. In all 9 of the homocolonial tests (condition 1), rescuers began by digging and, often, transporting sand away from the victim before they attempted to extricate the victim by limb pulling, which exposed the snare. Rescuers then were able to direct their behavior toward the snare itself, digging and transporting additional sand, as needed, to expose more of the snare, to which they returned again and again. These rescue behaviors can be seen in the two supporting video files (videos S1 and S2), attached to this paper. Not surprisingly, given the complete absence of rescue behavior in any but homocolonial tests (condition 1), a global comparison between the six conditions revealed a statistically significant difference in the duration of each of these four rescue behavior patterns (*P*<0.001, Kruskal - Wallis test; StatXact). Nonetheless, multiple comparisons between the six conditions using a sequential Bonferroni adjustment [20], showed that the duration of sand digging and snare biting, but not limb pulling or sand transport, was significantly greater toward the nestmate victim than toward any of the remaining test stimuli, namely an ant from a different colony (heterocolonial test), an ant belonging to a different species (heterospecific test), a larval cricket (prey test), or, in the two control tests, either a motionless (chilled) nestmate, or an empty snare apparatus (Wilcoxon-Mann-Whitney *U* test using Bonferroni's sequential adjustment method, α = 0.05). Neither condition 5 nor condition 6, both control conditions, elicited any behavior from ants and so are combined in Figure S1 (but, of course, not in any of the statistical analyses).

In stark contrast to the rescue behavior elicited by nestmates, *C. cursor* subjects were highly aggressive toward all other live test stimuli, except the motionless (chilled) nestmate (Figure S2), namely an ant from a different colony (heterocolonial test), an ant belonging to a different species (heterospecific test), or a larval cricket (prey test). Here, too, neither condition 5 nor condition 6, both control conditions, elicited any behavior from ants and so are combined in Figure S2 (but, again, were not combined in any of the statistical analyses). These aggressive behavior patterns, easily recognizable and observed in previous work with other species of *Cataglyphis* [19], [21], included threatening with open mandibles, formic acid projection (in which formic acid poison was sprayed in the direction of the test stimulus), dismemberment attempts, and biting. Although several rescue and aggressive behavior patterns involved the use of the mandibles, biting and attempts to dismember a body part were easily distinguished from limb pulling: In aggressive contexts, the gaster (abdomen) was always flexed, namely curved under the body, in preparation for formic acid spraying, whereas the gaster never was flexed during any form of rescue behavior. In addition, whereas aggressive biting and dismemberment attempts often were directed toward the victim's antennae, limb pulling never involved the antennae. *C. cursor* subjects never were aggressive toward their relatives.

A global comparison between the six conditions revealed a statistically significant difference in the duration of three of these four aggressive behavior patterns, namely threatening, biting and formic acid projection (*P*<0.001; Kruskal-Wallis test; StatXact). Nonetheless, multiple comparisons between the six conditions using a sequential Bonferroni adjustment [20], showed that, compared to a nestmate (homocolonial test) and both of the two control tests, none of which elicited any aggressive behavior, the duration of threatening was significantly greater toward an ant from a different colony (heterocolonial test), an ant belonging to a different species (heterospecific test), or a larval cricket (prey test), (Wilcoxon-Mann-Whitney *U* test using Bonferroni's sequential adjustment method, α = 0.05). In addition, for biting and formic acid projection, multiple comparisons showed that, compared to a nestmate (homocolonial test) and both of the two control tests, the duration of these behaviors was significantly greater toward an ant belonging to a different species (heterospecific test) (Wilcoxon-Mann-Whitney *U* test using Bonferroni's sequential adjustment method, α = 0.05). Dismembering attempts, which were rare, did not differ across the six conditions (Kruskal-Wallis test, *P* = 0.17).

Neither the motionless (chilled) nestmate, nor the empty snare elicited any of the eight behavioral reactions, either aggressive or rescue behavior, in any of the 9 tests each (i.e., 3 samples from each of 3 colonies) of these two conditions. Thus, the nylon snare in itself is not capable of eliciting snare biting or digging; moreover, an active nestmate must be caught in the snare to elicit such behavior.

## Discussion

In sum, our findings establish that, in *Cataglyphis cursor*, rescue behavior not only is directed exclusively toward nestmates but also the nestmate must be active. Thus, rescue behavior necessarily depends on some form of actively produced eliciting stimulus, already known to be a pheromone in several ant species [6], [9]–[11], but one that contains a component unique to each colony. In *C. cursor* ant colonies, as in many other hymenopteran societies, nestmates are close genetically because they are the progeny of a single queen (monogynous) and, thus, this form of nestmate discrimination can be an indirect mechanism for kin recognition. Moreover, *C. cursor* ants are able to engage in highly precise behavior directed toward the inanimate object that has entrapped their nestmate. Thus, our findings show that rescuers somehow were able to recognize what, exactly, held their relative in place and direct their behavior to that object in particular, demonstrating that rescue behavior is far more exact, sophisticated and complexly organized than previously observed. That is, limb pulling and digging behavior could be released directly by a chemical call for help and thus result from a relatively simple mechanism. However, it's difficult to see how this same simple releasing mechanism could guide rescuers to the precise location of the nylon thread, and enable them to target their bites to the thread itself.

## Materials and Methods

*C. cursor* ants were sampled from two colonies collected near Menerbes in 2006 and one colony near Bellegarde (both in Vaucluse, France) in 2005. In the laboratory, each of the three colonies was housed separately: A cylindrical closed nest box (15 cm diameter) was connected via a 20-cm plastic tube to an open foraging area, namely a plastic tray (28 cm×27.5 cm×8.5 cm high) covered with a thin layer of sand. Ants were fed mealworm larvae and an apple-honey mixture twice per week. The colony room was maintained at 28±2°C, 20 to 40% humidity, with a 12∶12 light: dark cycle. Two days prior to conducting the tests, all potential subject ants were chosen at random from both their nest box and accompanying foraging area, and individually marked on the thorax with a distinct spot of indelible paint (Uni Paint Marker PX 20®Mitsubishi Pencil Co., LTD). To conduct a test, a plastic ring (6.5 cm diameter×5.5 cm high), which was used to confine subjects for testing, was inserted firmly into the sand of the open foraging area, within 10 cm of the nest entrance, previously established as a marked area in *C. cursor* ants [22]. The ring wall was coated with fluon to prevent subject ants from escaping. Next, the test stimulus was prepared: A single nylon thread was inserted into a 1-cm-diameter round of filter paper, looped over the pedicel (waist) of the test stimulus (the “victim”), reinserted into the filter paper and pulled snugly to secure the test stimulus to the filter paper. Both ends of the thread were knotted to keep the test stimulus from escaping. The empty snare was prepared identically, leaving the same length of thread loop above the filter paper. Following preparation of the test stimulus, 5 previously marked subjects were chosen at random from a single colony and placed inside the plastic ring for 1–2 min, which allowed them to habituate to having been moved, as well as to the ring itself. We used the group of 5 nestmates per trial because preliminary tests showed that, to evoke rescue behavior, at least 5 nestmates must be present. Pilot work gave us good reason to believe that 5 nestmates may be the critical number to perceive and respond to a rescue situation. Next the filter paper, either containing a victim or left empty, was inserted in the centre of the ring and covered with a thin layer of sand, such that the head and thorax of each victim, or the empty loop, but not the filter paper, was visible. Following the 7-minute test, the test stimulus was removed and the plastic ring confining the subjects was lifted, permitting the ants to return to the nest box or to remain in the foraging area. No ant subject was tested twice. Tests were conducted during ants' active period, between 09:00 h and 14:00 h. A new nylon snare and filter paper were used for each test. Ant test stimuli also were marked so that they could be returned to their respective colonies and not used again.

Although, as described above, we used a group of 5 ants in each of the 54 tests, only 1–3 of the 5 nestmates in the homocolonial tests exhibited rescue behavior, with the number varying between tests. It was clear to us that not all ants are able to free the victim following the same algorithm. Thus, for each test, although the duration of each behavior was collected for each ant separately, we added the duration data across the 5 ants and analyzed the total duration of each behavior making the statistical unit the group of 5 ants. Because we did not find any statistical differences between the 3 different *C. cursor* test colonies for any of the 8 behavior patterns, either rescue or aggressive behavior (Kruskall-Wallis test, all *P*>0.55), we combined the results across the 3 colonies.

Statistical analysis of duration data was performed using StatXact 8, Cytel 2007 nonparametric tests. For global comparisons of K independent samples we used Kruskal-Wallis tests; for multiple comparisons between conditions we used Wilcoxon-Mann-Whitney U tests with Bonferroni's sequential adjustment method [20], α = 0.05.

## Supporting Information

Table S1 Operational definitions of rescue and aggressive behavior patterns.
(0.03 MB DOC)

Figure S1 Mean duration and S.E. of four rescue behavior patterns performed by n = 9 groups of 5 Cataglyphis cursor ants in response to an ensnared and partially buried test stimulus, which was either a nestmate (homocolonial), a member of another colony of C. cursor (heterocolonial), an ant from a different species (heterospecific), a prey item, or a control test stimulus, either an ensnared but motionless (chilled) nestmate or an empty snare, neither of which elicited any behavior.
(0.01 MB PDF)

Figure S2 Mean duration and S.E. of four aggressive behavior patterns performed by n = 9 groups of 5 Cataglyphis cursor ants in response to an ensnared and partially buried test stimulus, which was either a nestmate (homocolonial), a member of another colony of C. cursor (heterocolonial), an ant from a different species (heterospecific), a prey item, or a control test stimulus, either an ensnared but motionless (chilled) nestmate or an empty snare, neither of which elicited any behavior.
(0.02 MB PDF)

Video S1 Video rescue in ants
(38.60 MB AVI)

Video S2 Video of rescue in ants
(94.93 MB AVI)
