# Supplementary material for: Ants, Cataglyphis cursor, Use Precisely Directed Rescue Behavior to Free Entrapped Relatives
Source: PLoS One. 2009 Aug 12;4(8):e6573. doi: 10.1371/journal.pone.0006573 (PMC2719796; doi:10.1371/journal.pone.0006573)
Supplement: Table S1 — Operational definitions of rescue and aggressive behavior patterns. (0.03 MB DOC) [file pone.0006573.s001.doc]

| **Rescue behavior** | **Operational definitions** |
| --- | --- |
| Sand digging | Ant positions itself within 2 cm of the ensnared test stimulus and flicks sand backward, away from the test stimulus, using its anterior legs. |
| Limb-pulling | Ant grabs limb of test stimulus with mandibles and drags backwards with frequent antennation; gaster (abdomen) is *not* flexed. |
| Sand transport | Ant picks up sand particles with mandibles and moves it at least 5 mm, and as far as 2 cm, from the snare. |
| Snare biting | Ant bites and tugs at the nylon snare using mandibles. |
| **Aggressive behavior** | **Operational definitions** |
| Threatening | Ant approaches the test stimulus with mandibles opened to their maximally widest position; antennae are flexed backward, away from the test stimulus. |
| Dismemberment attempts | Ant grabs limb or antennae of test stimulus with mandibles and drags backwards; gaster (abdomen) is flexed, curved underneath the body, and antennae are flexed backward, away from the test stimulus. |
| Biting | Ant bites the limbs or body parts of the test stimulus; gaster (abdomen) is flexed, curved underneath the body. |
| Formic acid projection | Ant flexes its gaster (abdomen), curving it underneath its body, and projects formic acid in the direction of the test stimulus. |
