# Supplementary figures and images for: Ants, Cataglyphis cursor, Use Precisely Directed Rescue Behavior to Free Entrapped Relatives
Source: PLoS One. 2009 Aug 12;4(8):e6573. doi: 10.1371/journal.pone.0006573 (PMC2719796; doi:10.1371/journal.pone.0006573)

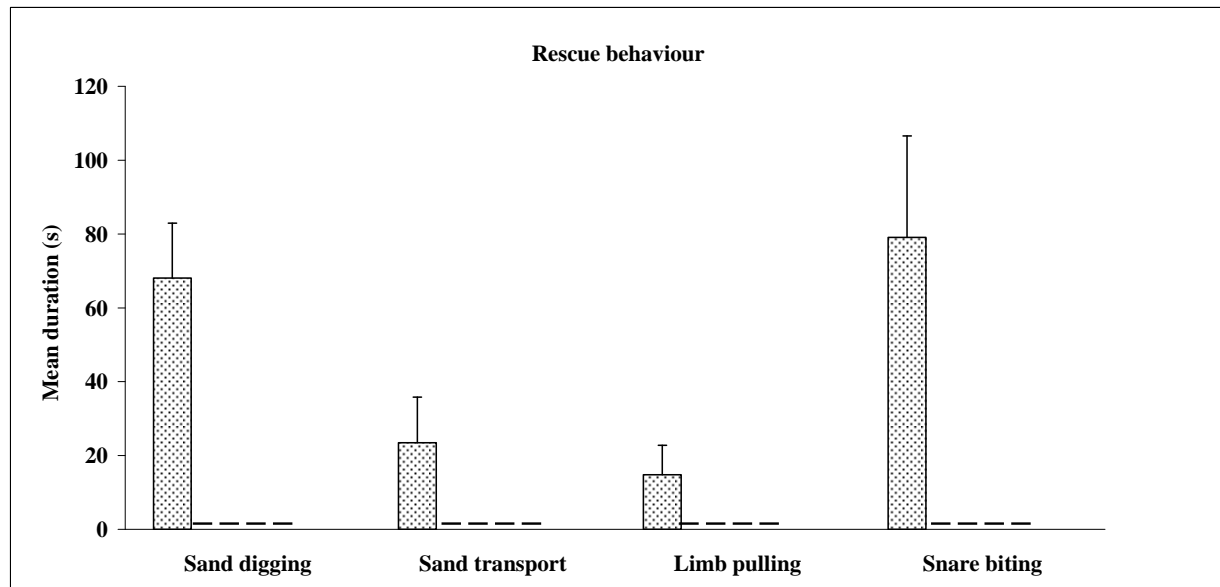

Homocolonial   Heterocolonial   Heterospecific   Prey   Controls

Supplement: Figure S1 — Mean duration and S.E. of four rescue behavior patterns performed by n = 9 groups of 5 Cataglyphis cursor ants in response to an ensnared and partially buried test stimulus, which was either a nestmate (homocolonial), a member of another colony of C. cursor (heterocolonial), an ant from a different species (heterospecific), a prey item, or a control test stimulus, either an ensnared but motionless (chilled) nestmate or an empty snare, neither of which elicited any behavior. (0.01 MB PDF) [file pone.0006573.s002.pdf]

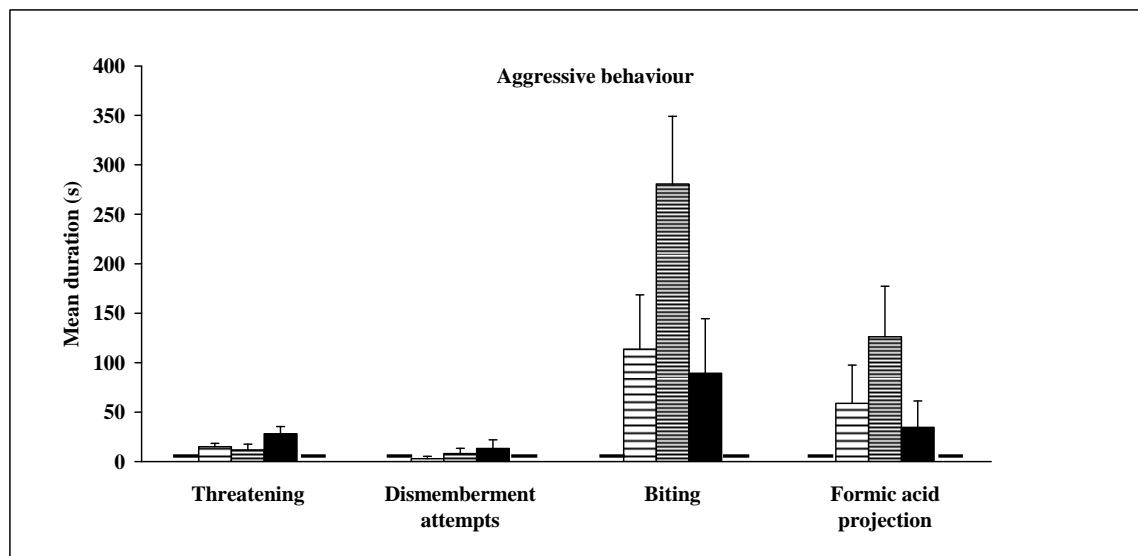

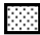 Homocolonial    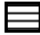 Heterocolonial    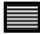 Heterospecific    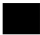 Prey    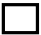 Controls

Supplement: Figure S2 — Mean duration and S.E. of four aggressive behavior patterns performed by n = 9 groups of 5 Cataglyphis cursor ants in response to an ensnared and partially buried test stimulus, which was either a nestmate (homocolonial), a member of another colony of C. cursor (heterocolonial), an ant from a different species (heterospecific), a prey item, or a control test stimulus, either an ensnared but motionless (chilled) nestmate or an empty snare, neither of which elicited any behavior. (0.02 MB PDF) [file pone.0006573.s003.pdf]
